# Concomitant presentation of eosinophilic or oncocytic mucoepidermoid carcinoma, immunoglobulin G4–related disease, and adult-onset asthma and periocular xanthogranuloma: Case report of 3 uncommon clinical entities

**DOI:** 10.1097/MD.0000000000030067

**Published:** 2022-08-12

**Authors:** Nikita Chhabra, John E. Cebak, Alessandra Schmitt, Devyani Lal, Allison C. Rosenthal, Cullen M. Taylor, Ryan M. Thorwarth, Ami A. Shah, Alicia Rodriguez-Pla

**Affiliations:** a Department of Neurology, Mayo Clinic School of Graduate Medical Education, Mayo Clinic College of Medicine and Science, Scottsdale, Arizona; b Division of Anatomic Pathology, Mayo Clinic, Scottsdale, Arizona; c Department of Otorhinolaryngology, Mayo Clinic, Phoenix, Arizona; d Division of Hematology and Medical Oncology, Mayo Clinic, Phoenix, Arizona; e Department of Otorhinolaryngology, Mayo Clinic School of Graduate Medical Education, Mayo Clinic College of Medicine and Science, Phoenix, Arizona; f Department of Ophthalmology, Mayo Clinic, Scottsdale, Arizona; g Division of Rheumatology, Mayo Clinic, Scottsdale, Arizona.

**Keywords:** adult-onset asthma and periocular xanthogranuloma, case report, IgG4-related disease, mucoepidermoid carcinoma, xanthelasma

## Abstract

**Rationale:**

Immunoglobulin (Ig) G4–related disease (IgG4-RD) reportedly has a strong relationship with adult-onset asthma and periocular xanthogranuloma (AAPOX) and may be linked to sclerosing mucoepidermoid carcinoma (MEC). We present a rare case of IgG4-RD and AAPOX occurring in a patient with resected eosinophilic or oncocytic MEC.

**Patient concerns:**

A 52-year-old woman was referred to our rheumatology clinic in 2020 to be evaluated for suspected IgG4-RD.

**Diagnoses:**

The patient had diagnoses of periorbital xanthelasmas, worsening glucocorticoid-dependent chronic rhinosinusitis and adult-onset asthma, and cervical lymphadenopathy persisting 2 years after resection of a low-grade MEC of a minor salivary gland.

**Interventions:**

Because the patient’s symptomatic relief was glucocorticoid dependent, IgG4-RD was suspected, and she was referred to our medical center. Her amylase and lipase levels were elevated. Serum IgG4 levels were initially within normal limits, but IgG4-RD was diagnosed because of the presence of lymphadenopathy and evidence of pancreatitis, which was shown on positron emission tomography/computed tomography. Furthermore, the IgG4 levels later increased without explanation. After the patient began combination therapy with a glucocorticoid (prednisone) and methotrexate, her symptoms improved but recurred when the daily oral glucocorticoid dosage decreased below 10 mg. An excisional biopsy of her right submandibular gland in 2021 yielded results consistent with IgG4-RD. In addition, AAPOX was diagnosed, given the presence of periocular edema and plaques, adult-onset asthma, and rhinosinusitis.

**Outcome:**

The patient was carcinoma free at last follow-up and was receiving medication to treat the other conditions.

**Lessons:**

The diagnosis of these 3 concomitant, uncommon entities required approximately 7 years of medical investigations. Clinicians should know that IgG4-RD, AAPOX, and MEC may occur together.

## 1. Introduction

Immunoglobulin (Ig) G4–related disease (IgG4-RD) is an autoimmune disease of lymphoplasmacytic infiltration and IgG4 deposition in multiple organ systems.^[1]^ Adult-onset asthma and periocular xanthogranuloma (AAPOX) is a rare non-Langerhans histiocytosis that commonly presents with the triad of periorbital swelling and xanthelasmas, adult-onset asthma, and chronic rhinosinusitis.^[2]^ A strong relationship has been reported between IgG4-RD and AAPOX.^[3]^

Primary malignant tumors of the salivary gland are rare. When they occur, most are mucoepidermoid carcinomas (MECs), which are composed of mucous, epidermoid, and intermediate cells. A rare variant known as sclerosing MEC also has unusual histologic characteristics consisting of abundant fibrous to hyalinized stroma and inflammatory cells.^[4]^ Sclerosing MEC is associated with increased numbers of IgG4-positive plasma cells, a finding that raises speculation about a potential link between MEC and IgG4-RD.^[5,6]^

Herein, we describe the case of a patient with a combination of these 3 uncommon clinical entities: IgG4-RD, AAPOX, and MEC. The variant of MEC, however, was the eosinophilic or oncocytic subtype (not the sclerosing subtype).

## 2. Case report

**Figure 1.**
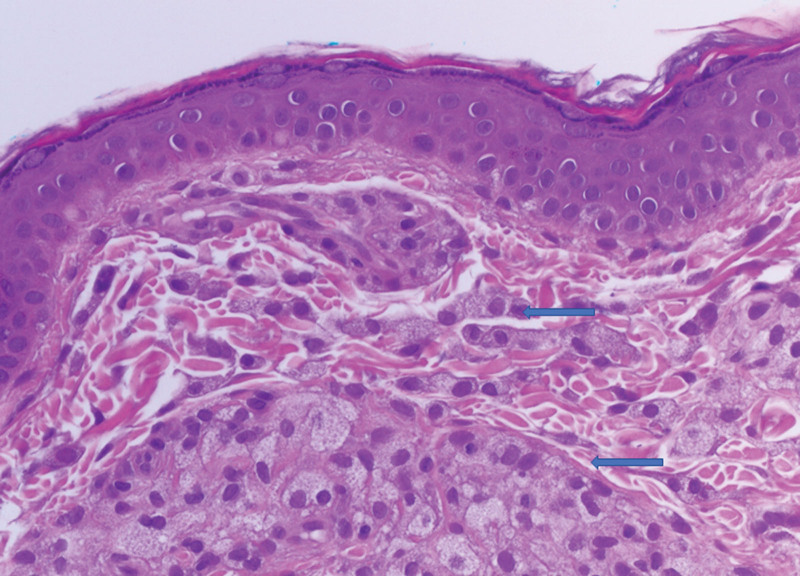
Shave biopsy specimen of the right lateral inferior pretarsal region. Xanthomatous infiltrate of the dermis (arrows; hematoxylin–eosin, original magnification ×400).

In 2020, a 52-year-old woman was referred to our rheumatology clinic by her otorhinolaryngologist to be evaluated for suspected IgG4-RD. In 2014, periorbital swelling and yellow plaques developed on her eyelids, and biopsy findings from her health care facility at that time were consistent with xanthelasma (Fig. 1). In 2015, she was diagnosed with adult-onset asthma and was prescribed an inhaler. The next year, after treatment of chronic rhinosinusitis with antibiotics and antihistamines was ineffective, she received a course of oral glucocorticoids. Glucocorticoid therapy substantially improved her symptoms of rhinosinusitis and asthma, greatly decreased the periorbital eyelid swelling, and minimized the periorbital plaques. Over the next 4 years, she resumed glucocorticoid treatment intermittently when her symptoms recurred.

**Figure 2.**
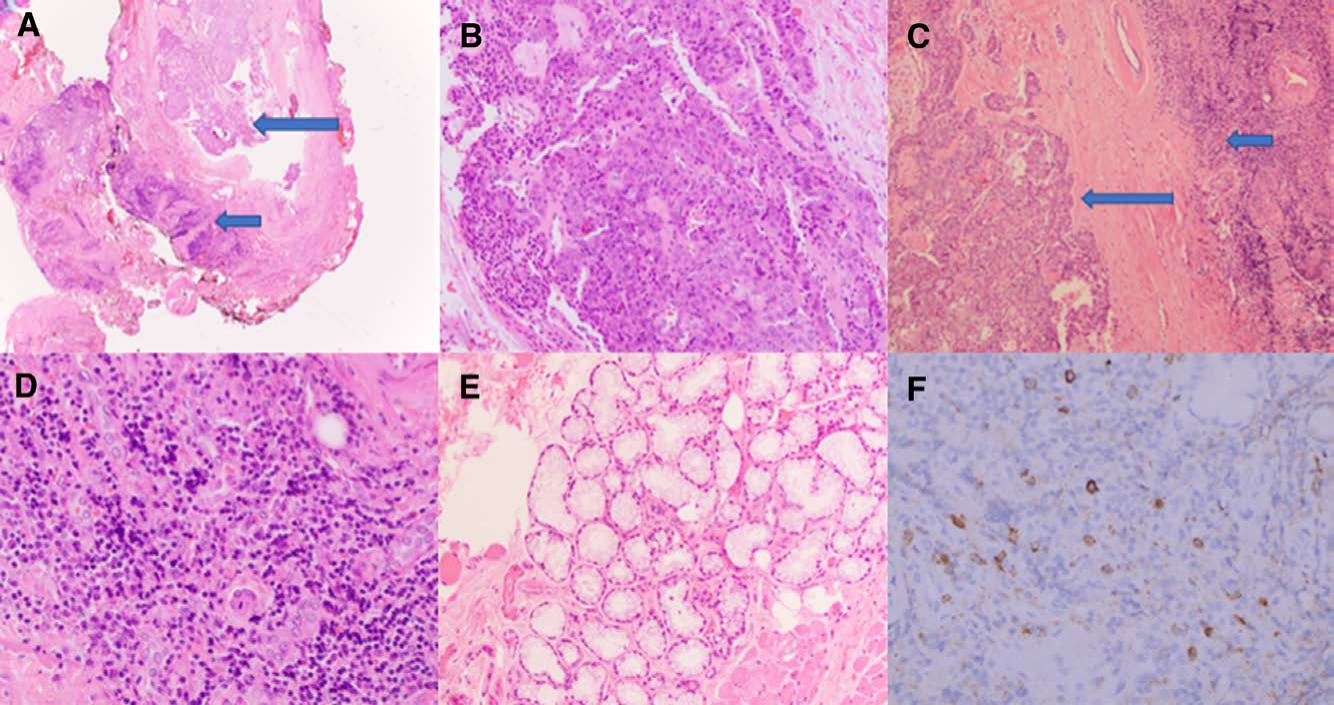
Pathologic findings of the MEC, oncocytic/eosinophilic variant. (A) Eosinophilic variant of MEC (long arrow) and peritumoral cuff of lymphocytes (short arrow; hematoxylin–eosin, original magnification ×20). (B) Eosinophilic variant of MEC (hematoxylin–eosin, original magnification ×200). (C) Eosinophilic variant of MEC (long arrow) and peritumoral cuff of lymphocytes (short arrow; hematoxylin–eosin, original magnification ×40). (D) Inflammatory infiltrate adjacent to the tumor (hematoxylin–eosin, original magnification × 400). (E) Minor salivary gland tissue away from the tumor (hematoxylin–eosin, original magnification ×200). (F) IgG4–positive plasma cells (original magnification ×400). Ig = immunoglobulin, MEC = mucoepidermoid carcinoma.

In 2017, the patient noticed cervical and submandibular lymphadenopathy and salivary gland swelling and underwent ultrasonography, which showed a tumor on the left side of the oral mucosa. The tumor was resected in 2018, and the pathologic findings were consistent with low-grade eosinophilic or oncocytic MEC of a minor salivary gland (Fig. 2A–E). A positron emission tomography/computed tomography (PET/CT) scan to evaluate for metastasis showed hypermetabolic cervical lymph nodes, soft-tissue hypermetabolism to the right of the thyroid gland, and pulmonary nodular ground-glass opacities, which were thought to be reactive. Also shown was a subcutaneous lesion in the patient’s right thigh with an associated inguinal lymph node. On biopsy, the cervical and inguinal lymph nodes were negative for cancer.

In 2020, the patient sought care at our institution because of persistent cervical lymphadenopathy, worsening chronic rhinosinusitis, inhaler-refractory asthma, recurrence of the orbital swelling, and more pronounced periorbital plaques. Results of initial examinations by an otorhinolaryngologist and an allergist were inconclusive, but IgG4-RD was suspected. The previous pathologic slides of the resected oral cavity tumor in the left retromolar trigone/cheek were reviewed, and additional IgG4 and CD138 immunohistochemical stains were performed to evaluate for underlying IgG4-related sclerosing disease (Fig. 2F). The tumor cells were positive for cytokeratin 5/6, and in the mucous cells, the intracytoplasmic mucin was highlighted by a mucicarmine stain. Results of tests for smooth muscle actin, S-100 protein, and γ-globulin were normal. Up to 40 IgG4-positive plasma cells per high-power field surrounded the tumor. As a marker of total plasma cells, CD138 showed the percentage of IgG4-positive cells at 90%. The final diagnosis was low-grade eosinophilic or oncocytic MEC. IgG4 induction by the adjacent MEC remained possible,^[7]^ but given the patient’s unique constellation of symptoms, IgG4-RD could not be excluded from the diagnosis. She was then referred to our rheumatology clinic for further evaluation.

**Figure 3.**
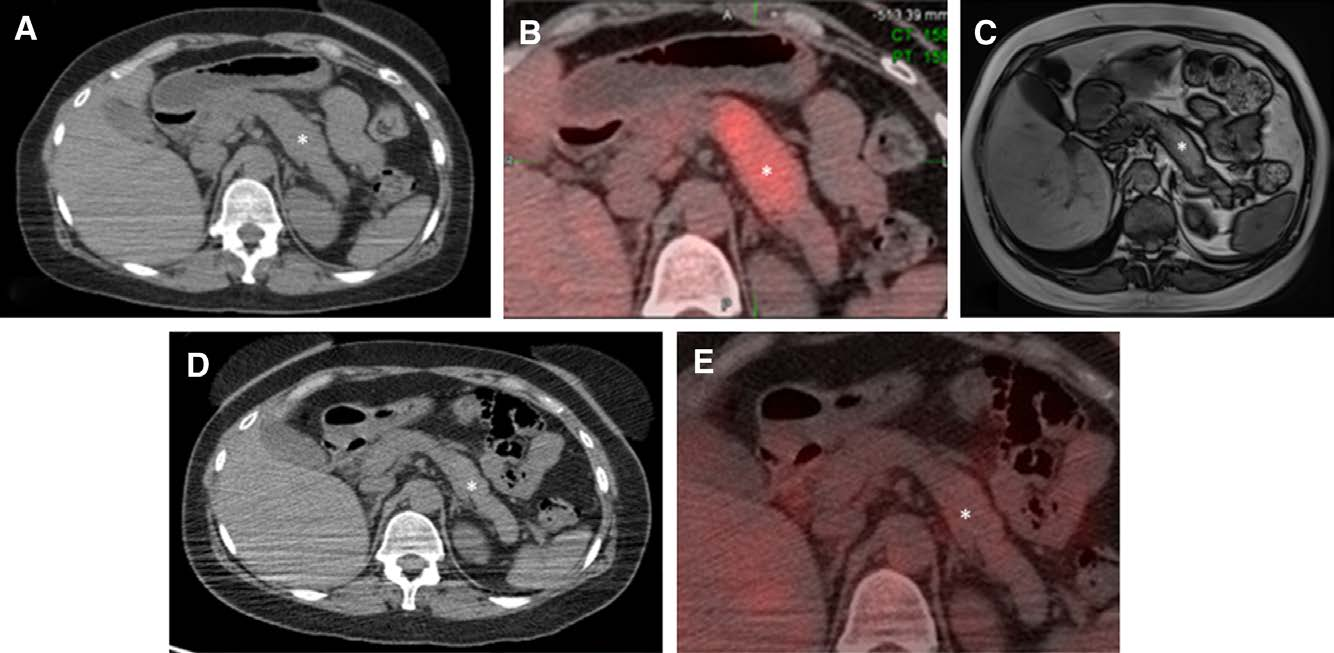
Images of pancreatitis. (A) PET/CT scan from October 2020 showing pancreatic swelling consistent with pancreatitis. (B) PET scan from October 2020 showing standardized uptake values of 6.6 to 8.1 in the pancreas. (C) T1-weighted magnetic resonance image of the abdomen acquired in the axial plane in December 2020. The pancreas overall appears smaller in diameter than that on PET/CT performed in October 2020 (A), consistent with resolving pancreatitis. (D), PET/CT scan from June 2021 showing near resolution of diffuse pancreatic swelling. (E) PET scan from June 2021 showing decreased pancreatic standardized uptake values (2.3–3.3). Asterisks indicate pancreatitis. CT = computed tomography, PET = positron emission tomography.

Results of the rheumatologic workup on October 27, 2020, showed an initially normal IgG4 level of 38.8 mg/dL (Table 1). A repeated PET/CT scan showed interval development of diffuse swelling of the pancreas (Fig. 3A) with increased tracer uptake consistent with pancreatitis (Fig. 3B), a common finding in IgG4-RD.^[8]^ Amylase and lipase levels were elevated at 130 and 68 mg/dL, respectively. The patient, however, did not have symptoms of pancreatitis.

**Table 1:** Results of inflammatory markers and Ig levels.

Laboratory test	Reference range	October 2020	January 2021
Inflammatory markers			
ESR, mm/h	0–29.0	35.0	26.0
CRP, mg/L	≤8.0	6.4	4.0
Ig levels, mg/dL*			
IgE, kU/L	≤214.0	75.3	Not done
IgA	61.0–356.0	180.0	130.0
IgM	37.0–286.0	96.0	97.0
IgG	767.0–1590.0	1820.0	610.0
IgG1	341.0–894.0	1070.0	324.0
IgG2	171.0–632.0	486.0	149.0
IgG3	18.4–106.0	183.0	50.5
IgG4	2.4–121.0	38.8	139.0

We reviewed the previous pathologic slides of the eyelid for the right lateral inferior pretarsal region, where microscopic evaluation of the hematoxylin–eosin–stained sections showed a shave biopsy specimen that extended through the superficial dermis. Interstitial nests of xanthomatized histiocytes were present in the dermis, and several multinucleated xanthoma cells with scattered single plasma cells and lymphocytes were identified; histopathologic findings were consistent with xanthelasma (Fig. 1). Although cases of IgG4-RD with bronchial asthma and chronic rhinosinusitis have been reported,^[9]^ the presence of xanthelasma led us to a probable diagnosis of AAPOX, which has been reported in relation to IgG4-RD.^[3,10]^ In our patient, however, the substantial lymphocytic infiltrates, fibrosis, and Touton giant cells often described in AAPOX were not seen. The infiltrate consisted almost entirely of lipid-laden histiocytes, and the numbers of plasma cells were insufficient for definitive IgG4 testing. A second eyelid biopsy was performed at our institution for clinical correlation because of variability due to tissue sampling, and results showed xanthomatous infiltrate involving the dermis—again consistent with xanthelasma.

Despite the lack of characteristic histopathologic findings of AAPOX, we still thought that the patient had IgG4-RD and AAPOX, given her clinical characteristics, and decided to offer her rituximab treatment, which has been used to treat IgG4-RD^[11,12]^ and IgG4-RD coexisting with AAPOX.^[13]^ The patient, however, declined rituximab therapy because she was concerned about prolonged immunosuppression during the coronavirus disease 2019 pandemic.^[14]^ She was started instead on an oral glucocorticoid regimen (prednisone) and methotrexate, which have proved useful for treating both IgG4-RD and AAPOX.^[2,15]^ Subsequently, she noted substantial improvement in her symptoms. Additionally, abdominal magnetic resonance imaging performed 2 months after initiation of treatment showed resolving pancreatitis (Fig. 3C). A subsequent measurement of her IgG4 level in January 2021 was elevated without explanation (Table 1). Another PET/CT scan in June 2021 showed resolution of tracer-avid findings at the left parotid fossa, left pharyngeal region, and bilateral aspect of the neck. These images also showed mild to borderline residual uptake in the right inguinal region, skin of the right thigh, and near resolution of diffuse pancreatic swelling (Fig. 3D) with standardized uptake values of 2.3 to 3.3 (Fig. 3E), a decrease from prior values of 6.6 to 8.1.

**Figure 4.**
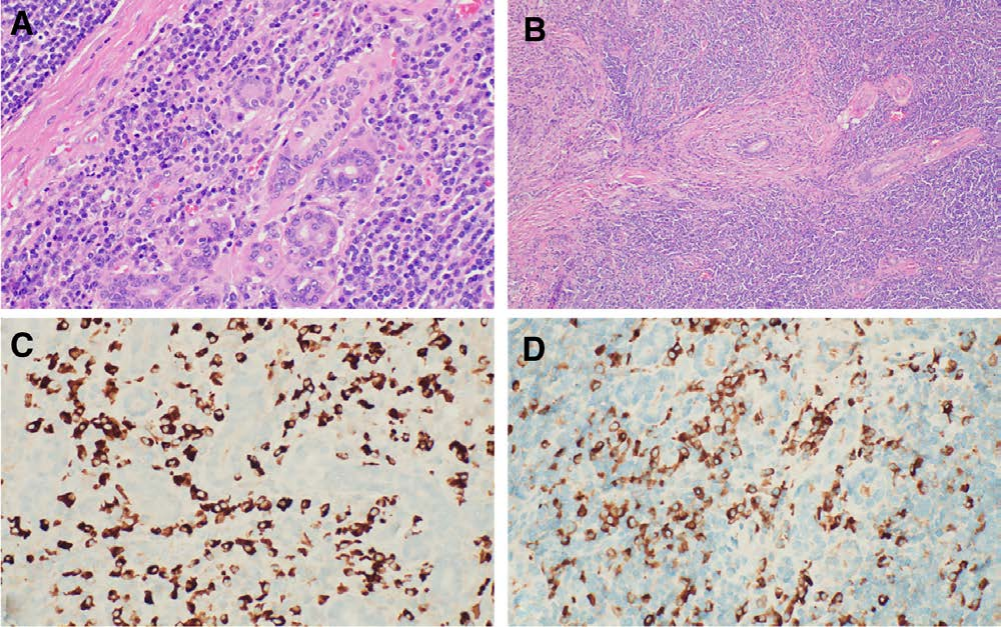
Biopsy specimen of a right submandibular gland. (A) Findings showing a dense lymphoplasmacytic infiltrate and fibrous bands (H&E, original magnification ×400). (B) Submandibular gland is diffusely infiltrated by dense lymphoplasmacytic inflammatory infiltrate with storiform fibrosis (H&E, original magnification ×100). (C) Numerous Ig G4 cells stained positive with IHC stain (original magnification ×400). (D), IHC-positive plasma IgG cells (original magnification ×400). H&E = hematoxylin–eosin, Ig = immunoglobulin, IHC = immunohistochemical.

**Figure 5.**
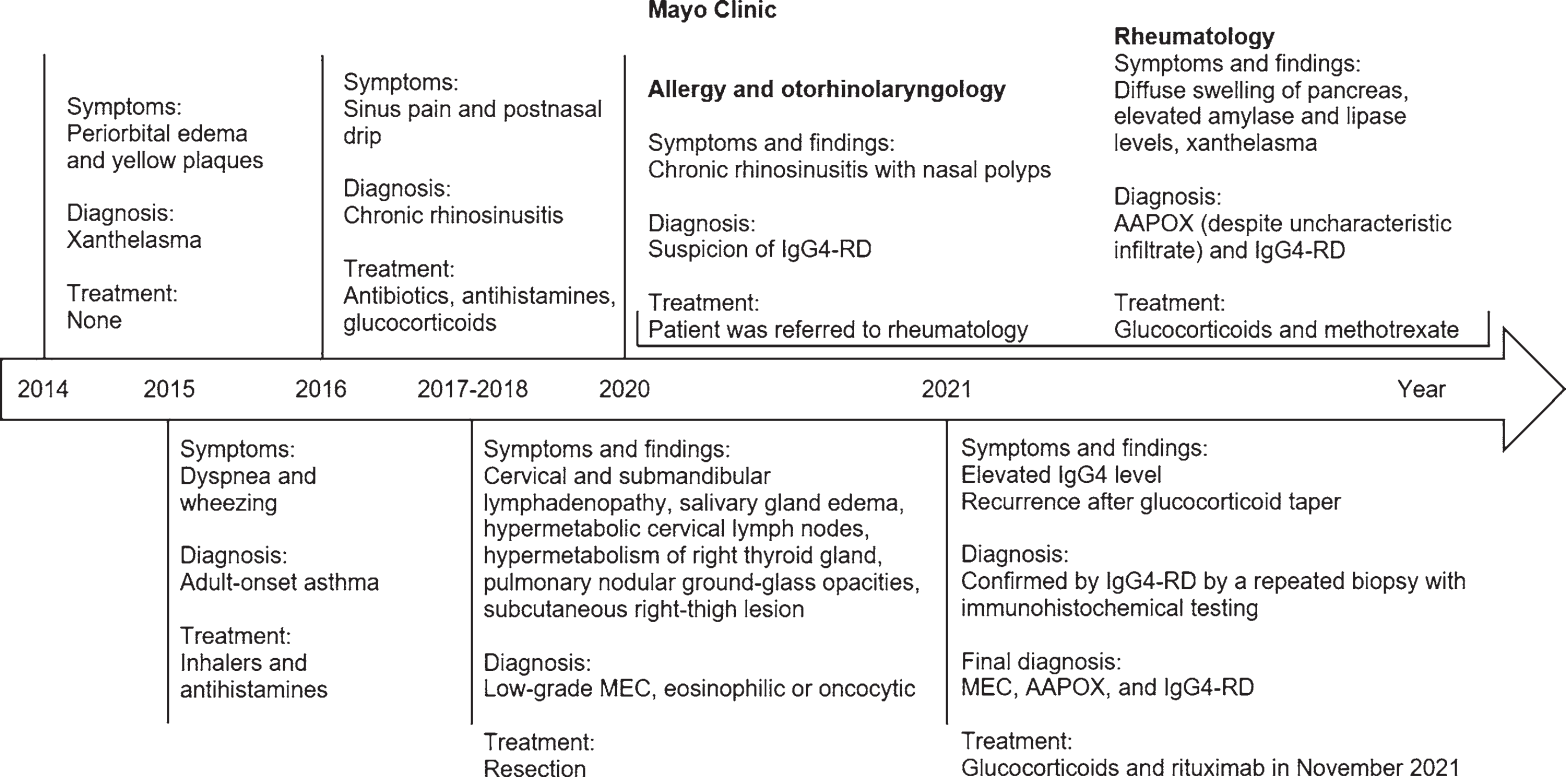
Timeline of the case. AAPOX = adult-onset asthma and periocular xanthogranuloma, IgG4 = immunoglobulin G4, IgG4-RD = IgG4-related disease, MEC = mucoepidermoid carcinoma.

When the patient tried to taper the dose of glucocorticoids, symptoms recurred despite combination therapy with methotrexate and glucocorticoid. Because liver enzyme levels became elevated with higher doses of methotrexate, the methotrexate dose had to be decreased. In September 2021, to confirm the diagnosis of IgG4-RD, an excisional biopsy of a right submandibular gland was performed after the patient had stopped glucocorticoid and methotrexate therapy for a few weeks. The biopsy specimen showed patchy involvement of the submandibular gland by a dense chronic fibroinflammatory process, with chronic lymphoplasmacytic inflammation accompanied by storiform fibrosis and dense fibrous bands. Although no definite obliterative phlebitis was seen, immunohistochemical findings for IgG and IgG4 showed markedly elevated IgG4-positive plasma cells (up to 180/high-power field) and an elevated IgG4:IgG ratio (up to 70%). Additional immunohistochemical tests for CD3, CD5, CD10, CD20, CD21, CD43, BCL2, BCL6, and cyclin D1 were performed, along with in situ hybridization of κ and λ light chains. Results of these studies highlighted a mixed infiltrate of small T cells and small B cells without aberrant antigen expression, along with a polytypic population of plasma cells without light chain restriction, offering no evidence of an underlying lymphoproliferative disorder. No evidence of neoplasia or cancer was seen (Fig. 4A–D). After a multidisciplinary discussion and in agreement with the patient, we restarted glucocorticoid treatment and planned to start treatment with rituximab. The timeline for the patient’s case, with relevant data on symptoms, diagnoses, and treatments, is shown in Figure 5.

## 3. Discussion

We report a rare case of IgG4-RD and AAPOX occurring in a patient with resected eosinophilic or oncocytic MEC, which was confirmed by review of the pathology slides obtained from the initial treating physicians. In patients with cancer, IgG4-positive cells may occur as an inflammatory response, a finding that raises the suspicion of MEC-mediated IgG4 production.^[7,16]^ The mechanism behind this inflammatory response is thought to be cytokine-mediated, involving interleukin-10 and transforming growth factor β, both of which induce increased IgG4-positive cells and fibrosis.^[17]^ Tian et al^[5]^ reported several cases of sclerosing MEC with surrounding IgG4-positive cells, but none of their patients had IgG4-RD in other organs or in the nontumoral salivary gland.

Malignant tumors have been reported in patients with IgG4-RD. Gill et al^[18]^ reported elevated serum IgG4 levels and salivary duct carcinoma of the parotid gland in a patient with chronic sclerosing sialadenitis and regional lymphadenopathy. Tasaki et al^[6]^ described a patient with sclerosing MEC who had eosinophils in the submandibular gland along with IgG4-related chronic sclerosing inflammation and who postoperatively had an elevated serum IgG4 level. The authors suggested that the chronic inflammatory process might have triggered the sclerosing MEC to develop, but they could not exclude the possibility that the prominent IgG4-positive plasma cell and fibrosis were immune reactions caused by the tumor.^[6]^ Our patient had a different type of MEC, called eosinophilic or oncocytic variant. In the resected tumor specimen, IgG4-positive cells were increased only surrounding the tumor, a finding that could be related just to the presence of the tumor^[7]^ but does not exclude IgG4-RD from the diagnosis. Furthermore, our patient had systemic organ involvement, including pancreatitis and rhinosinusitis, which are both common in IgG4-RD,^[11,12,19]^ but rhinosinusitis also commonly occurs in AAPOX. Although the patient’s serum IgG4 level was not initially high, ultimately the patient’s IgG4 level became elevated, for which we have no explanation, and results of a salivary gland biopsy were consistent with IgG4-RD.

The presence of periorbital edema, adult-onset asthma, and rhinosinusitis led to the diagnosis of AAPOX, a rare non-Langerhans histiocytosis^[20]^ characterized by periorbital disease with a specific granulomatous inflammation composed of foamy histiocytes and Touton giant cells.^[21]^ The findings from our patient’s eyelid biopsies were consistent with xanthelasma, although the typical histopathologic findings of AAPOX were not observed, which could be related to tissue sampling. AAPOX is part of a rare heterogeneous group of disorders that includes 3 other clinical syndromes: adult-onset xanthogranuloma, necrobiotic xanthogranuloma, and Erdheim–Chester disease. For our patient, a PET/CT scan excluded the poorer prognosis of Erdheim-Chester disease, wherein long bone lesions predominate,^[3]^ and her symptoms suggested a diagnosis of AAPOX. London et al^[10]^ reported that substantial histopathologic similarities and clinical overlap exist between AAPOX and IgG4-RD, and they later suggested a strong relationship between the 2 conditions.^[3]^ This previously reported relationship between IgG4-RD and AAPOX in addition to our patient’s constellation of symptoms makes the coexistence of both diagnoses very likely in this case.

The patient’s symptoms substantially improved with combination therapy (oral glucocorticoid and methotrexate). The decision regarding starting methotrexate treatment required a risk-benefit conversation with the patient, given her history of MEC. The potential for liver toxicity with higher doses of methotrexate and the recurrence of the patient’s symptoms after decreasing the methotrexate dose led us to confirm the IgG4-RD diagnosis with a salivary gland biopsy. After confirmation, we restarted treatment with glucocorticoids and are planning to start rituximab therapy.^[8,11]^

A limitation of our report is that it is a single case study. Although we cannot conclude from a single case that a link exists between IgG4-RD, AAPOX, and MEC, authors of previous reports suggested a possible association between salivary gland tumors and IgG4-RD when patients have atypical symptoms. Reports of additional cases and case series may help clarify whether a true association exists between IgG4-RD, AAPOX, and MEC or whether their occurrence in a single patient is merely coincidental. However, clinicians should know that IgG4-RD, AAPOX, and MEC may occur together. In this unusual case, the road to diagnosis and implementation of an effective, long-term treatment for this patient took 7 years.

The Mayo Clinic Institutional Review Board does not require approval for a single case report. The patient’s written consent was obtained for the purpose of publication.

## Author contributions

Nikita Chhabra, DO: Substantial contribution to the acquisition of data, drafting of the manuscript, and revising it critically for important intellectual content and final approval; agree to be accountable for all aspects of the work in ensuring that questions related to the accuracy or integrity of any part of the work are appropriately investigated and resolved

John E. Cebak, DO, PhD^a^: Substantial contribution to the acquisition of data, drafting of the manuscript, and revising it critically for important intellectual content and final approval; agree to be accountable for all aspects of the work in ensuring that questions related to the accuracy or integrity of any part of the work are appropriately investigated and resolved

Alessandra Schmitt, MD^b^: Substantial contribution to the acquisition of data, drafting of the manuscript, and revising it critically for important intellectual content and final approval; agree to be accountable for all aspects of the work in ensuring that questions related to the accuracy or integrity of any part of the work are appropriately investigated and resolved

Devyani Lal, MD^c^: Substantial contribution to the acquisition of data, revising the manuscript critically for important intellectual content and final approval; agree to be accountable for all aspects of the work in ensuring that questions related to the accuracy or integrity of any part of the work are appropriately investigated and resolved

Allison C. Rosenthal, DO^d^: Substantial contribution to revising the manuscript critically for important intellectual content and final approval; agree to be accountable for all aspects of the work in ensuring that questions related to the accuracy or integrity of any part of the work are appropriately investigated and resolved

Cullen M. Taylor, MD^e^: Substantial contribution to revising the manuscript critically for important intellectual content and final approval; agree to be accountable for all aspects of the work in ensuring that questions related to the accuracy or integrity of any part of the work are appropriately investigated and resolved

Ryan M. Thorwarth, MD^e^: Substantial contribution to revising the manuscript critically for important intellectual content and final approval; agree to be accountable for all aspects of the work in ensuring that questions related to the accuracy or integrity of any part of the work are appropriately investigated and resolved

Ami A. Shah, MD^f^: Substantial contribution to revising the manuscript critically for important intellectual content and final approval; agree to be accountable for all aspects of the work in ensuring that questions related to the accuracy or integrity of any part of the work are appropriately investigated and resolved

Alicia Rodriguez-Pla, MD, PhD^s^: Substantial contribution to revising the manuscript critically for important intellectual content and final approval; agree to be accountable for all aspects of the work in ensuring that questions related to the accuracy or integrity of any part of the work are appropriately investigated and resolved

## Acknowledgments

The Scientific Publications staff at Mayo Clinic provided substantive editing, proofreading, administrative, and clerical support.

## Footnotes

* Unless otherwise indicated.
